# Supplementary material for: A central arctic extreme aerosol event triggered by a warm air-mass intrusion
Source: Nat Commun. 2022 Sep 8;13:5290. doi: 10.1038/s41467-022-32872-2 (PMC9458659; doi:10.1038/s41467-022-32872-2)
Supplement: Supplementary file 1 — Supplementary Information [file 41467_2022_32872_MOESM1_ESM.pdf]

Supplementary information for:

A central arctic extreme aerosol event triggered by a warm air-mass intrusion

Lubna Dada[1,12]*, Hélène Angot[1], Ivo Beck[1], Andrea Baccarini[1], Lauriane L. J. Quéléver[2], Matthew Boyer[2], Tiia Laurila[2], Zoé Brasseur[2], Gina Jozef[3,4,5], Gijs de Boer[3,6,7], Matthew D. Shupe[3,6], Silvia Henning[8], Silvia Bucci[9], Marina Dütsch[9], Andreas Stohl[9], Tuukka Petäjä[2], Kaspar R. Daellenbach[10], Tuija Jokinen[2,11] & Julia Schmale[1]*

[1]Extreme Environments Research Laboratory, Ecole Polytechnique Fédérale de Lausanne (EPFL) Valais Wallis, 1951 Sion, Switzerland.
[2]Institute for Atmospheric and Earth System Research, INAR/Physics, Faculty of Science, University of Helsinki, 00014 Helsinki, Finland.
[3]Cooperative Institute for Research in Environmental Science, University of Colorado, Boulder, CO 80309, USA.
[4]National Snow and Ice Data Center, University of Colorado, Boulder, CO 80309, USA.
[5]Department of Atmospheric and Oceanic Sciences, University of Colorado Boulder, Boulder,CO,USA.
[6]Physical Sciences Laboratory, National Oceanic and Atmospheric Administration, Boulder,CO80305, USA.
[7]Integrated Remote and InSitu Sensing,University ofColorado, Boulder,CO80309,USA.
[8]Leibniz Institute for Tropospheric Research, Permoserstrasse 15, 04318 Leipzig, Germany.
[9]Department of Meteorology and Geophysics, University of Vienna, 1090 Vienna, Austria.
[10]Laboratory of Atmospheric Chemistry, Paul Scherrer Institute, 5232 Villigen, Switzerland.
[11]Climate & Atmosphere Research Centre (CAREC), the Cyprus Institute, P.O. Box 27456 Nicosia 1645, Cyprus.
[12]Present address: Laboratory of Atmospheric Chemistry, Paul Scherrer Institute, 5232 Villigen, Switzerland.
*Correspondence to: lubna.dada@epfl.ch and julia.schmale@epfl.ch

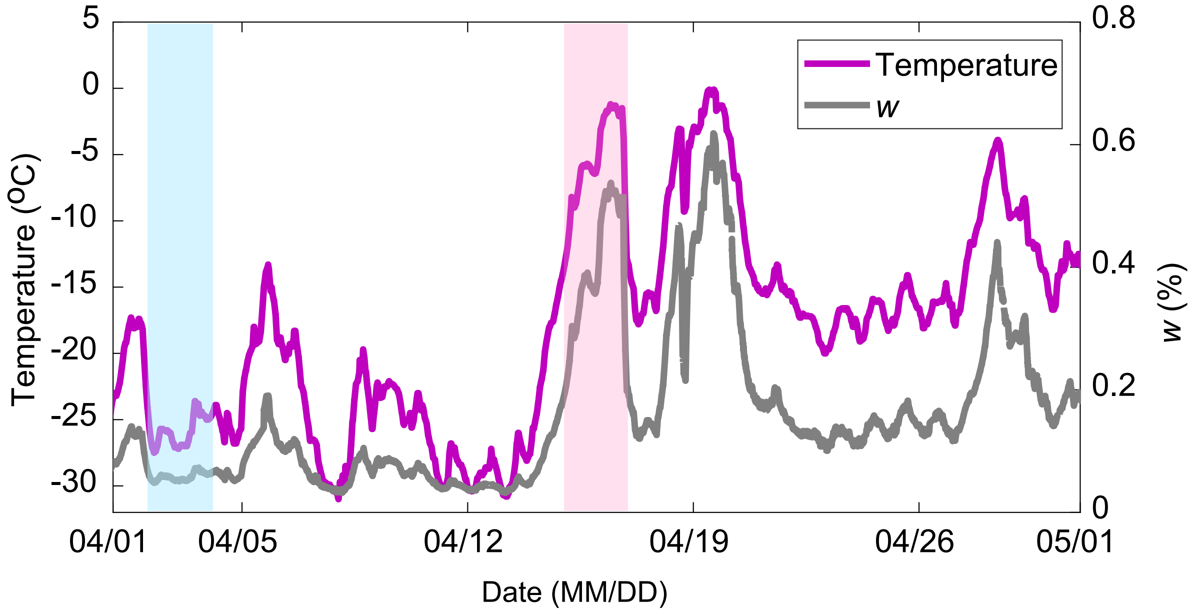

Supplementary Figure 1. Temperature and atmospheric water vapor (*w*). Temperature at 29 m (left) measured by the Polarstern weather station and water vapor (right) measured in the Swiss container by a Picarro during April 2020. The shaded areas represent the periods of focus of this study, 'background' conditions in blue, and warm intrusion period in pink. The intrusion observed on April 19th is not discussed further in this study as the period is affected by local precipitation and ship pollution.

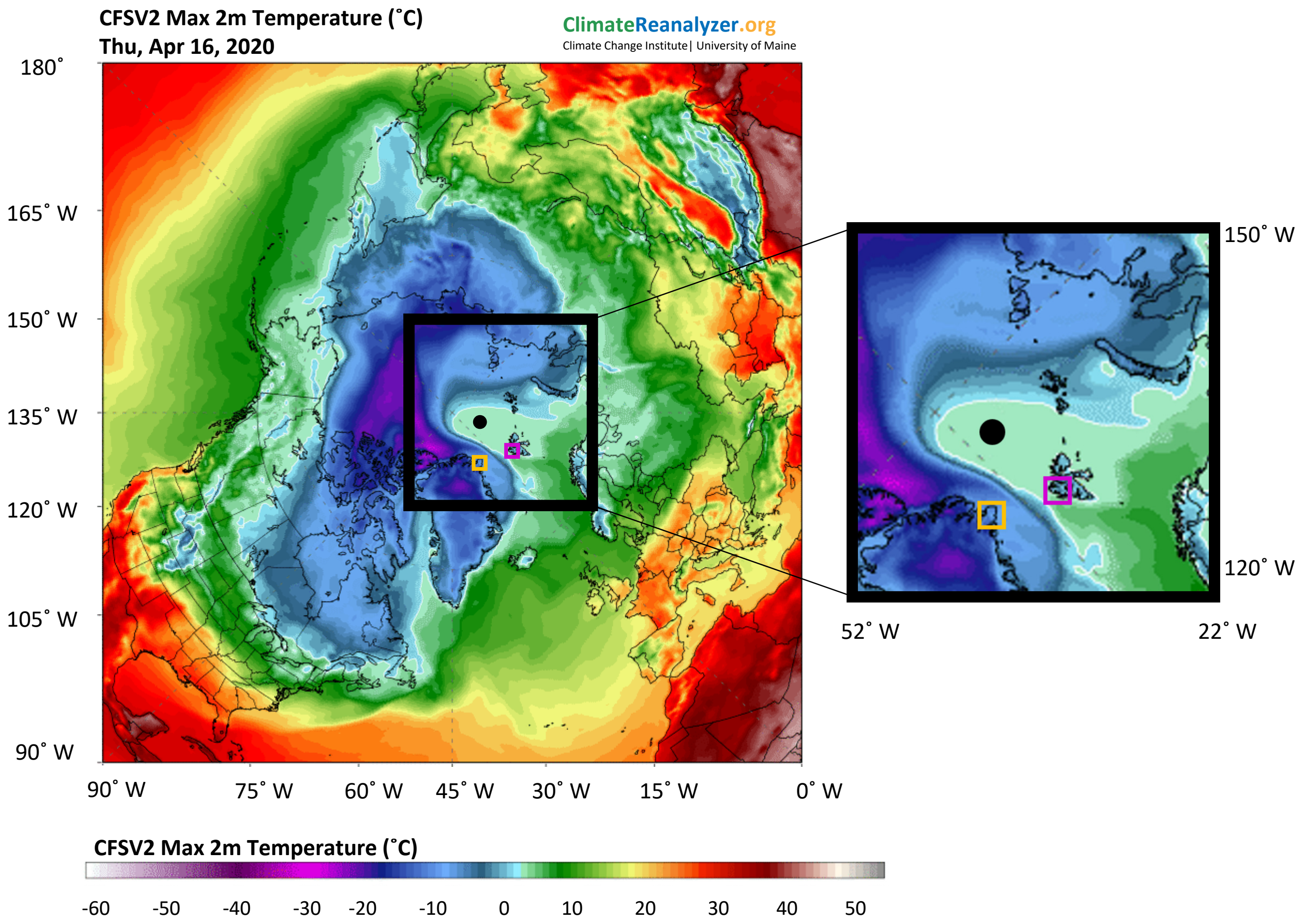

Supplementary Figure 2. Extent of the warm intrusion. The heat map shows the near-surface temperature in the Arctic on April 16th 2020 during the second peak of the warm air-mass intrusion event. The intrusion affects a large area of the central Arctic. The figure also shows that ground-based Arctic stations, for instance Villum research station at Station Nord (81.36° N, 16.40 °W) , Greenland is not affected by the intrusion, while the Ny-Ålesund observatories (78.55 °N, 11. 56 °E) are. Image from Climate Reanalyzer (https://ClimateReanalyzer.org, last access on 13.12.2021), Climate Change Institute, University of Maine, USA. The location of Polarstern (84.34° N, 13.09° E) is marked with a black circle and those of Villum and Ny-Ålesund are marked with yellow and magenta squares, respectively.

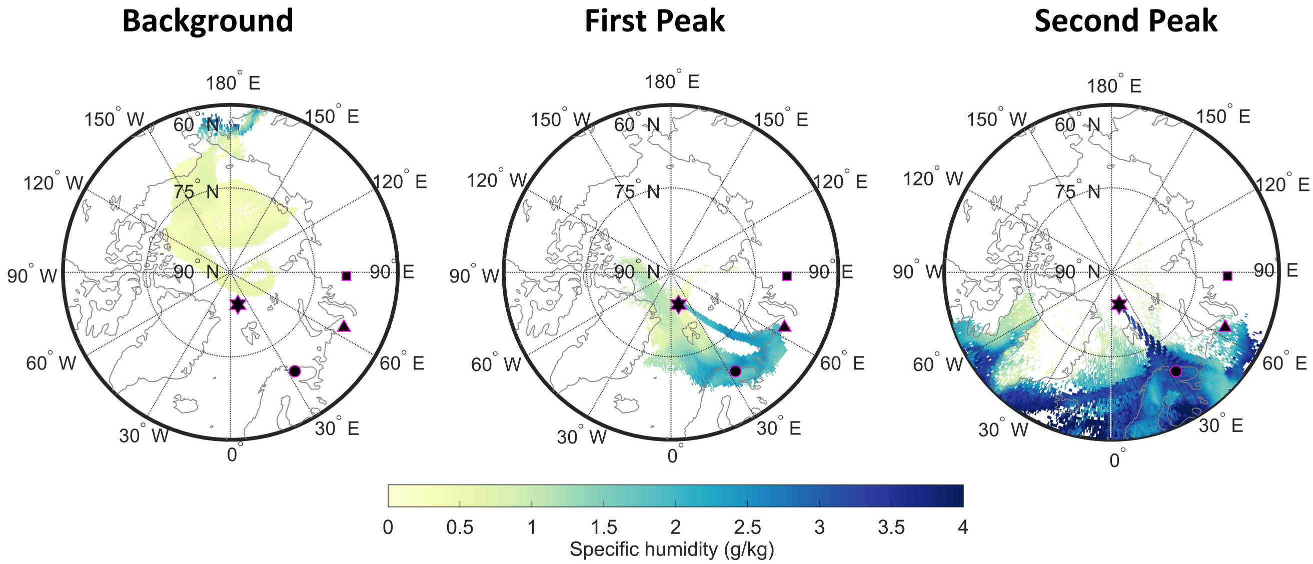

Supplementary Figure 3. Specific humidity of air-masses. Specific humidity of the air mass with simulated particles residing below 100 m altitude, obtained from 7-day backward simulations with FLEXPART during the background period (left panel), first peak of the intrusion event (middle panel) and second peak of the intrusion event (right panel). The position of Polarstern is marked with a star, that of Norilsk (69.3558° N, 88.1893° E) with a square, that of Vorkuta (67.4969° N, 64.0602° E) with a triangle, and that of Murmansk (68.9733° N, 33.0856° E) with a circle. Polarstern drifted from 84.95° N, 14.99° E on 02.04.2020 at 12:00 to 84.34° N, 13.09° E on 16.04.2020 at 12:00.

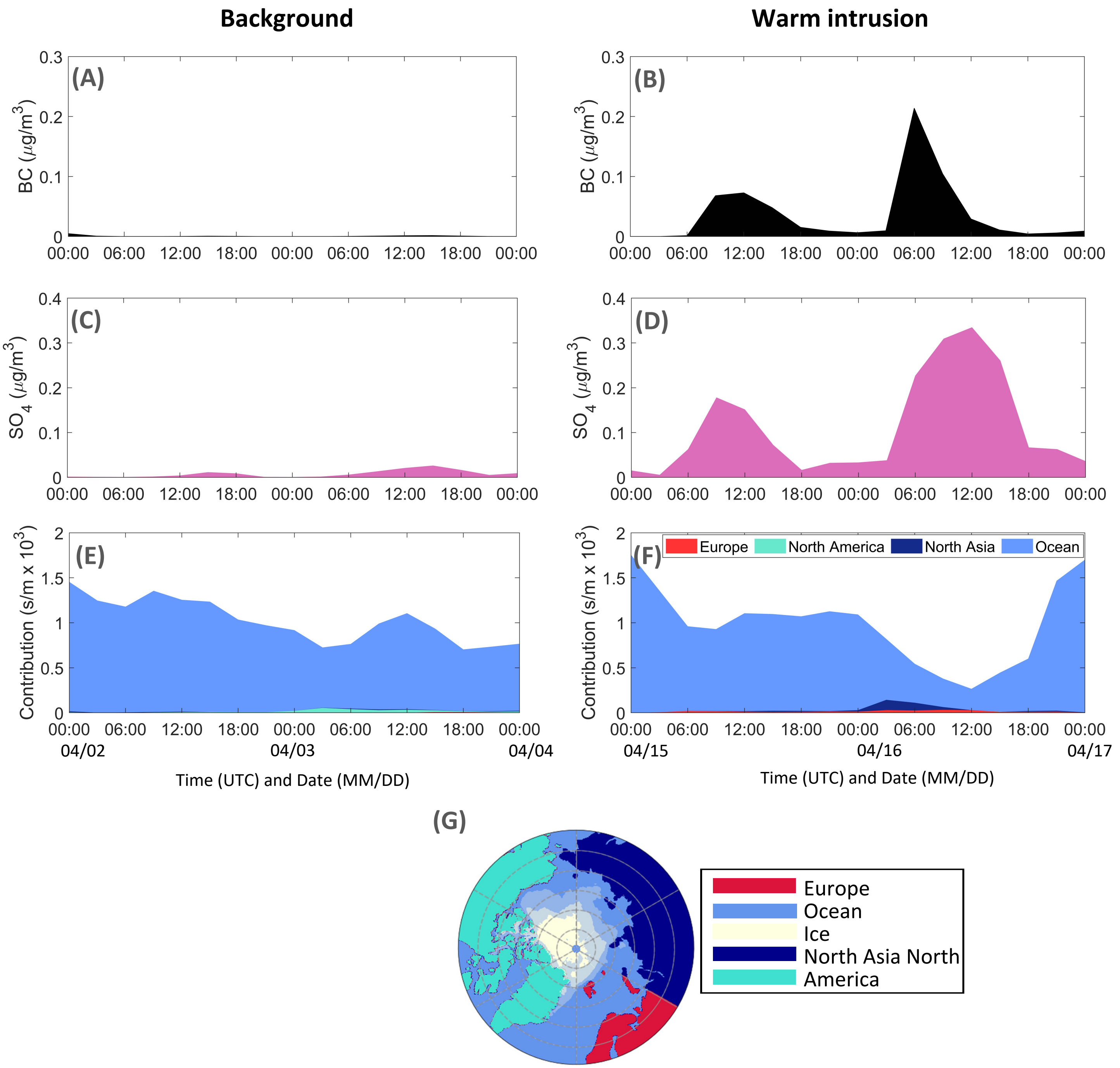

Supplementary Figure 4. Source contributions of black carbon and sulfate. Time series of regional source contributions integrated over the whole domain for BC during (A) the background on April 2nd – 3rd and (B) intrusion period on April 15-16th. Time series of source contributions integrated over the whole domain for the FLEXPART SO_4 aerosol tracer during (C) the background on April 2nd – 3rd and (D) intrusion period on April 15-16th. Regional surface contribution (representative of the relative contribution of each sub region) during (E) the background on April 2nd – 3rd and (F) intrusion period on April 15-16th. (G) Map of regions used in (E) and (F). The units, s/m are

equivalent to the concentration in μg/m3 that one would obtain if each region would have an emission flux of 1μg/m^2/s everywhere. It is important to note here that although emissions from the ocean appear to contribute most, they are a proxy for the adjacent location. For example, the ocean north of Eurasia is a proxy for the emissions in Eurasia.

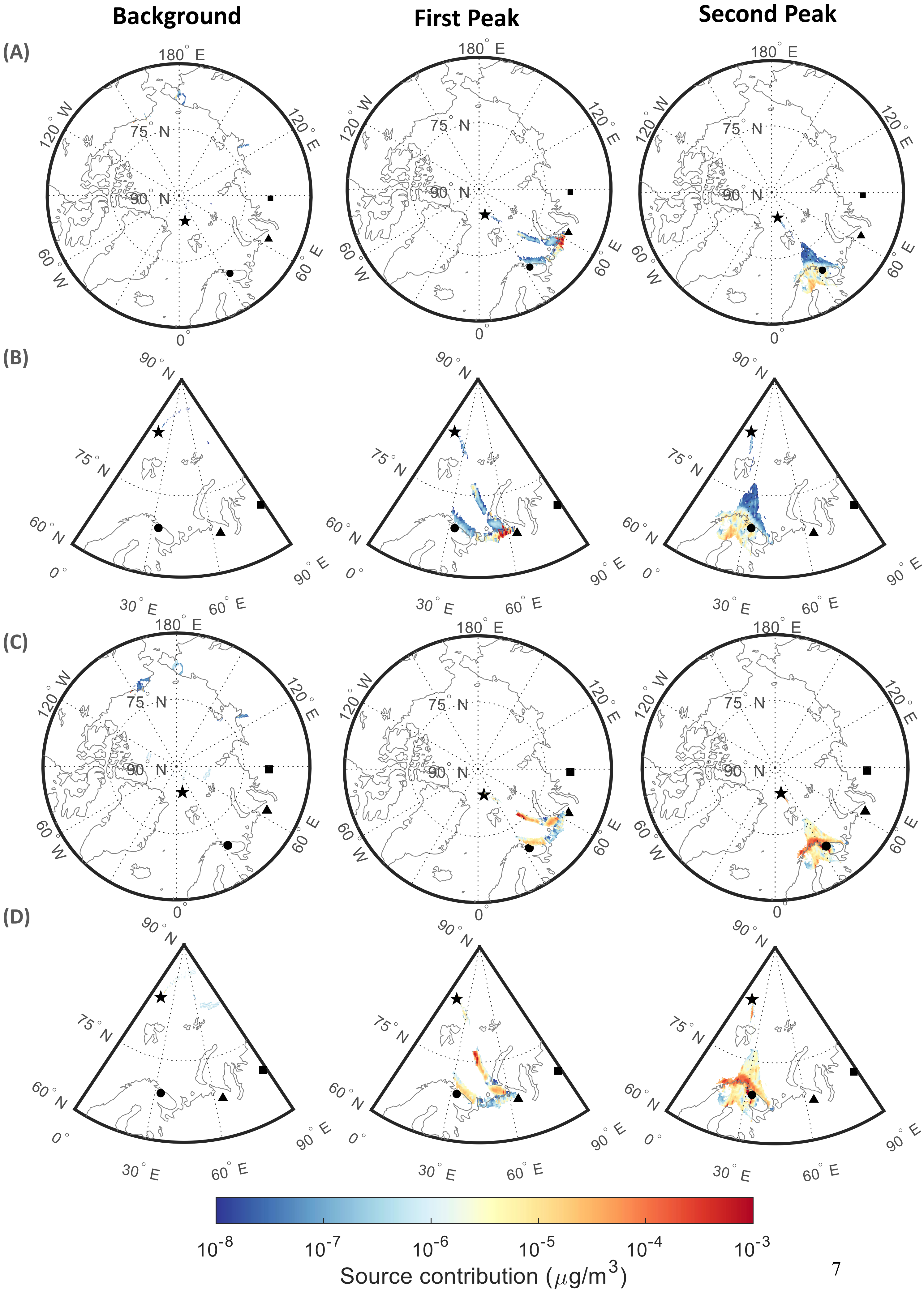
Background
First Peak
Second Peak
(A)
(B)
(C)
(D)
180° E
120° W
120° E
75° N
90° N
60° W
60° E
0°
90° N
75° N
60° N
0°
90° E
30° E
60° E
10^-8
10^-7
10^-6
10^-5
10^-4
10^-3
Source contribution (μg/m^3)

Supplementary Figure 5. Black carbon and sulfate footprint source contributions. (A) Black carbon source contribution (below 100 m a.g.l.), and inserts shown in (B), for a passive air tracer obtained from 7-day backward simulations with FLEXPART during the background period (left panel), first peak of the intrusion event (middle panel) and second peak of the intrusion event (right panel). (C) Sulfate footprint source contribution (below 100 m a.g.l.), and inserts shown in (D), for a passive air tracer obtained from 7-day backward simulations with FLEXPART during the background period (left panel), first peak of the intrusion event (middle panel) and second peak of the intrusion event (right panel). The position of Polarstern is marked with a star, that of Norilsk (69.3558° N, 88.1893° E) with a square, that of Vorkuta (67.4969° N, 64.0602° E) with a triangle, and that of Murmansk (68.9733° N, 33.0856° E) with a circle. Polarstern drifted from 84.95° N, 14.99° E on 02.04.2020 at 12:00 to 84.34° N, 13.09° E on 16.04.2020 at 12:00.

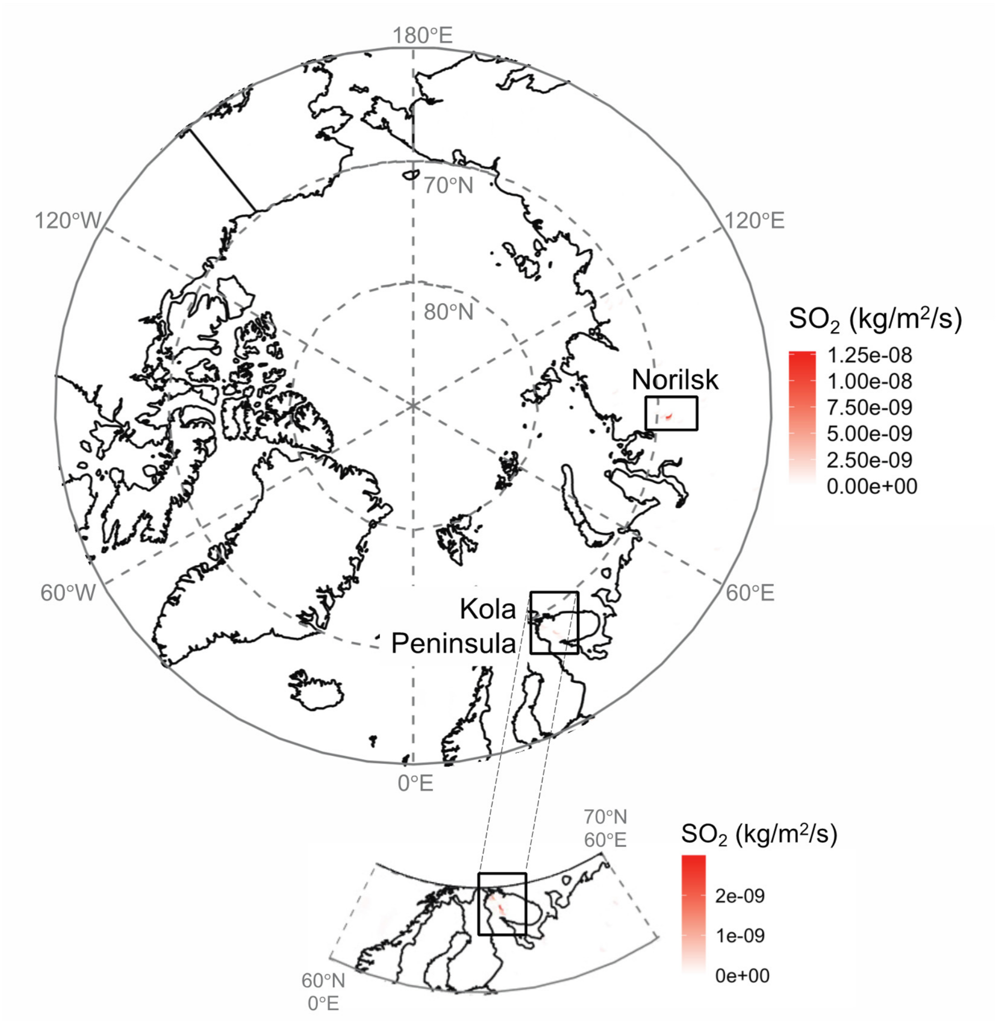

Supplementary Figure 6. SO_2 emission flux. Mean SO_2 emission flux (in kg/m^2/s) according to the Community Emissions Data System (CEDS) global anthropogenic emission inventory for year 2017 from McDuffie et al.[1]. This figure highlights two emission hotspots: the Norilsk (69.3558° N, 88.1893° E) smelting facility in Siberia and the metallurgical industry in the Kola Peninsula, north-west Russia.

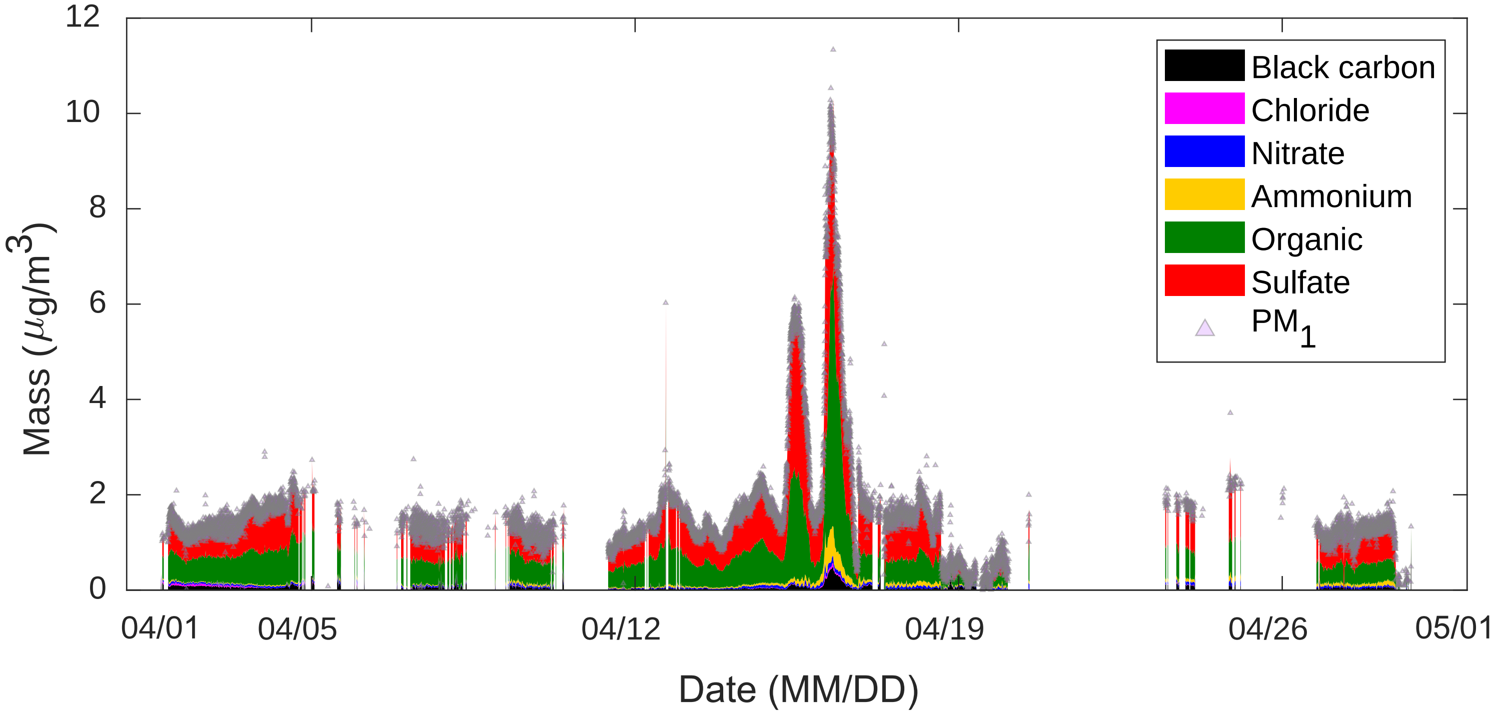

Supplementary Figure 7. Particulate matter speciated mass composition. Non-refractory particulate matter with diameter smaller than 1 µm and black carbon ($NR\text{-}PM_1$ + BC) speciated mass composition averaged over 15 min during periods not affected by ship exhaust during April 2020 (see Methods subsection development of pollution mask for AMS data). Grey triangles are the $NR\text{-}PM_1$ + BC in 1.5 min time resolution.

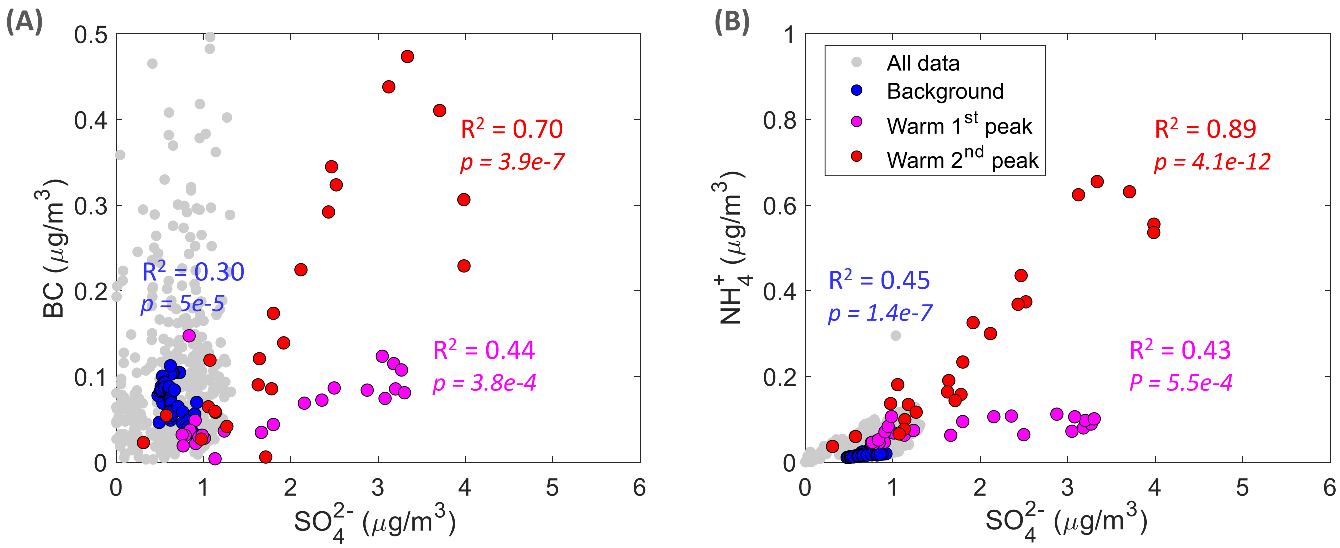

Supplementary Figure 8. Correlation between sulfate, black carbon and ammonium. Correlation between SO_4^{2-} and (A) BC and (B) NH_4^+. Data averaged for 1 hour. “All data” refers to all data obtained during April 2020. Correlation coefficients and statistical significance (p-values) are shown in the figure.

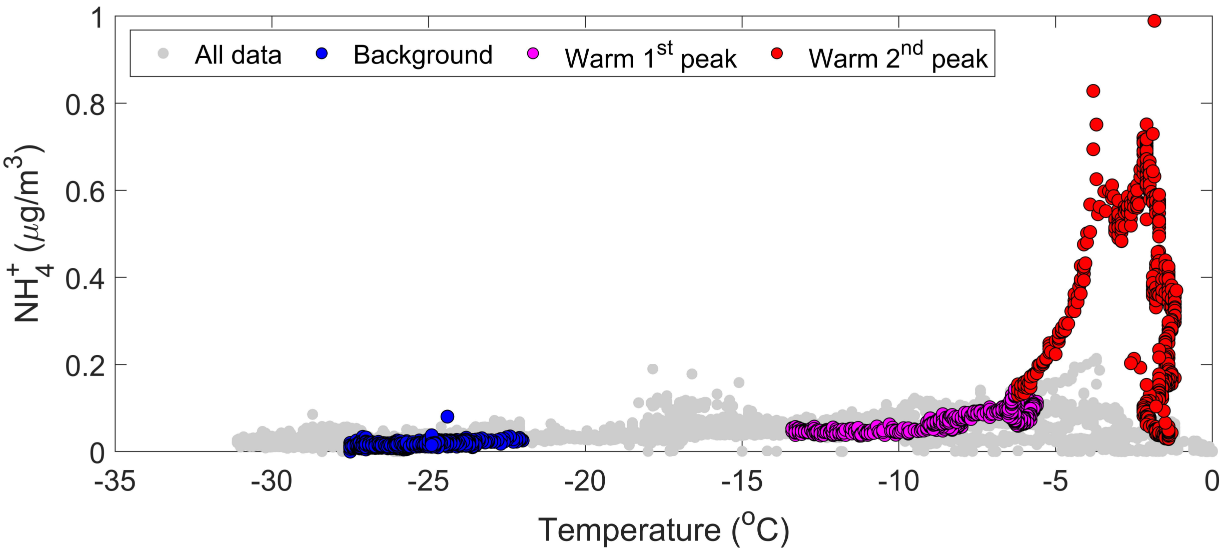

Supplementary Figure 9. Evolution of ammonium as a function of temperature. Data are in 1.5 min time resolution (time resolution of the HR-AMS). "All data" refers to all data obtained during April 2020.

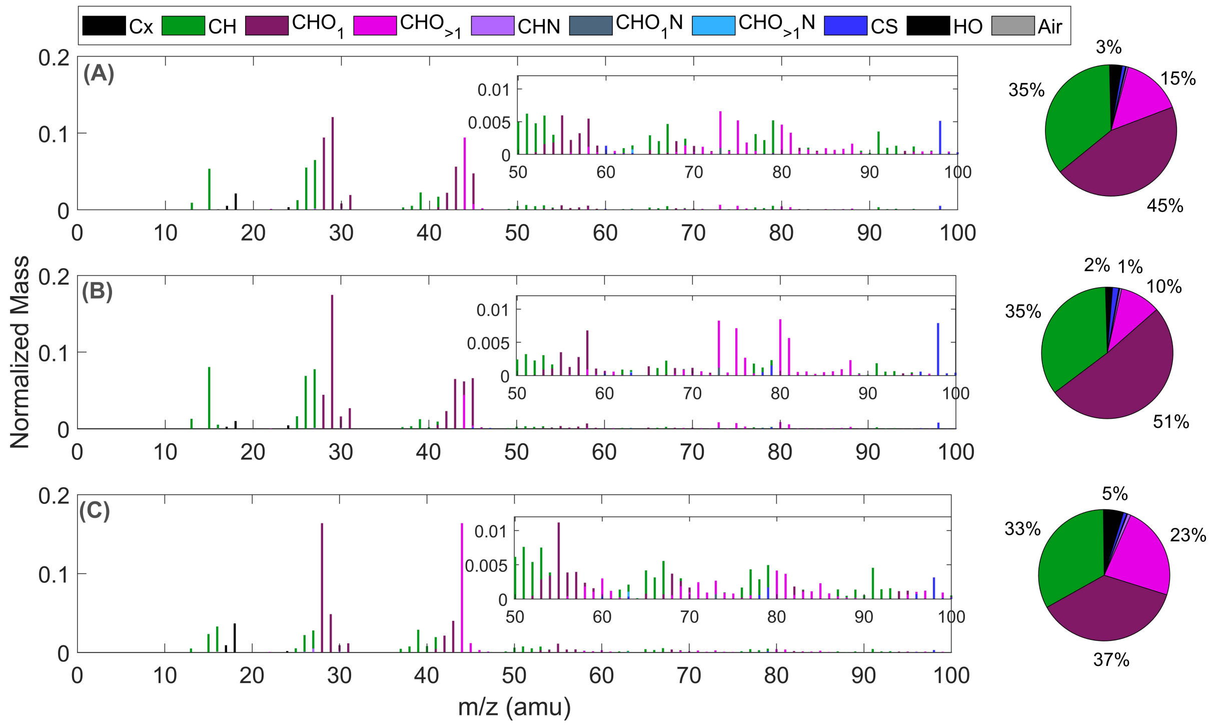

Supplementary Figure 10. Organic aerosol composition during the background and warm intrusion event. Average mass spectra normalized to sum of all peaks and pie charts showing the contributions of each family to total organic (Org) aerosol for the different periods (A) background, (B) first peak of the intrusion, and (C) second peak of the intrusion. Inserts are between m/z 50 and 100. Generally, it is expected that the organics are more oxygenated the longer they have resided in the atmosphere[2] due to photochemical processing. Hence, the Arctic haze aerosol should in theory be more oxygenated unless there is less solar irradiance, yet, the mean global radiation is 57.31 and 67.26 W/m^2, during the background and intrusion period, respectively[3]. Given that there is no significant difference, other mechanisms such as heterogeneous processing seem to be more important to explain the difference. This is particularly true for the difference in oxygenation between the two intrusion peaks. The occurrence of heterogeneous processing is plausible, because the warm and moist intrusion was accompanied by substantial cloud formation. More oxygenated Orgs are also more hygroscopic and hence better cloud condensation nuclei.

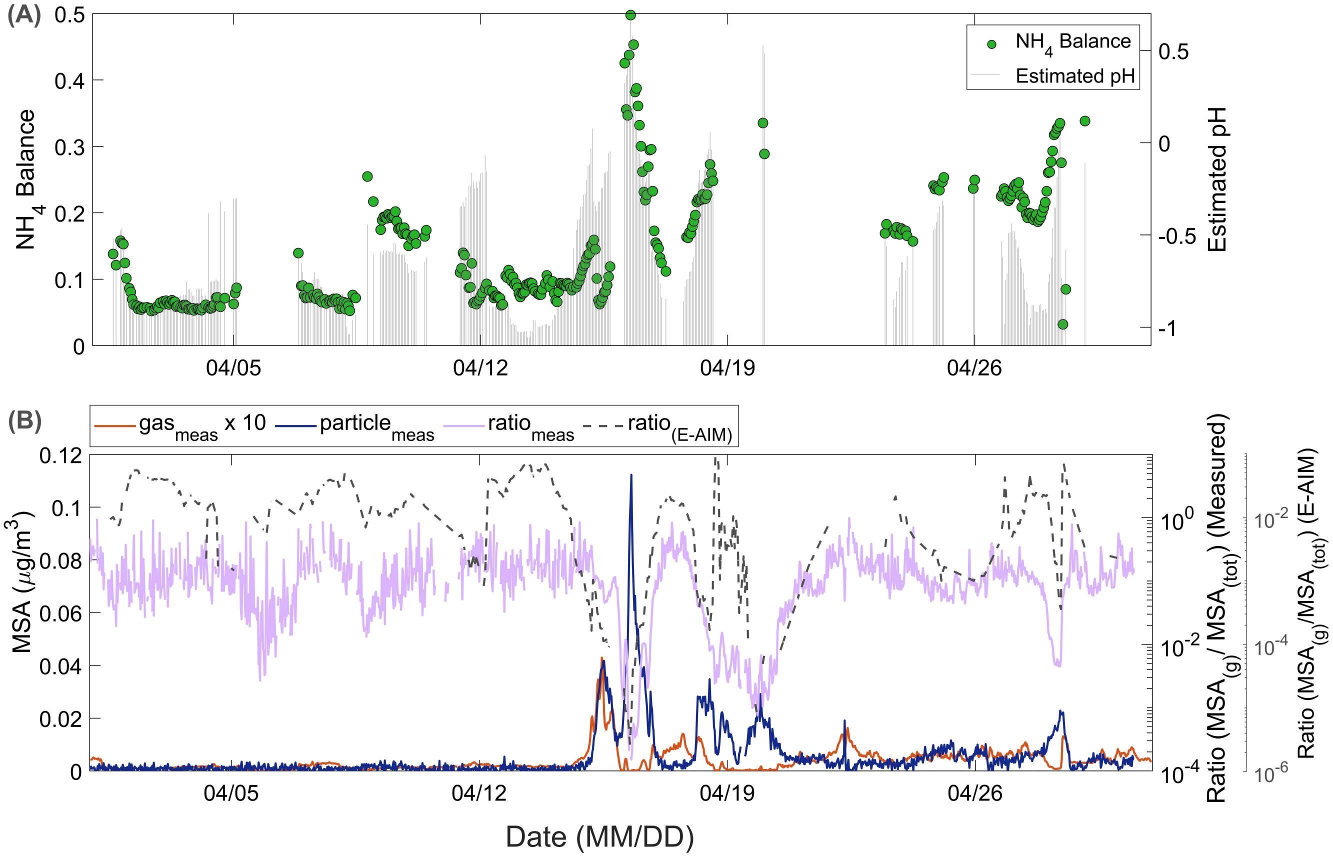

Supplementary Figure 11. Aerosol acidity and MSA partitioning. (A) Estimated acidity based on ammonium balance (green dots, left axis) and estimated aerosol pH calculated using E-AIM model (bars, right axis) (B) Gas phase methane sulfonic acid (MSA) measured using NO_3-CI-APiTOF in µg/m^3 (gas$_{meas}$; orange solid line, left axis – multiplied by 10 for visual aid), particle phase MSA measured using AMS (particle$_{meas}$; blue solid line, left axis) and the ratio of gas MSA$_g$ to MSA$_p$ (right axis), from measurements (lila solid line) and based on E-AIM model calculations (gray dashed line). E-AIM results during ship pollution periods are filtered out.

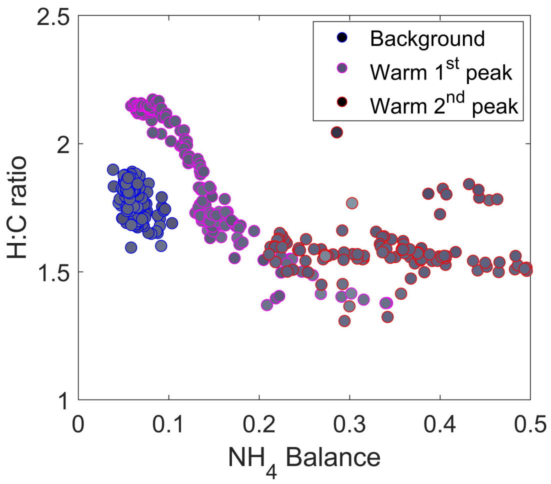

Supplementary Figure 12. Composition of organics as a function of aerosol acidity.

Hydrogen -to-carbon (H:C) ratio as a function of aerosol acidity estimated by NH_4^+ balance.

Note that during the plotted periods, the oxygen -to-carbon ratio is around 0.8.

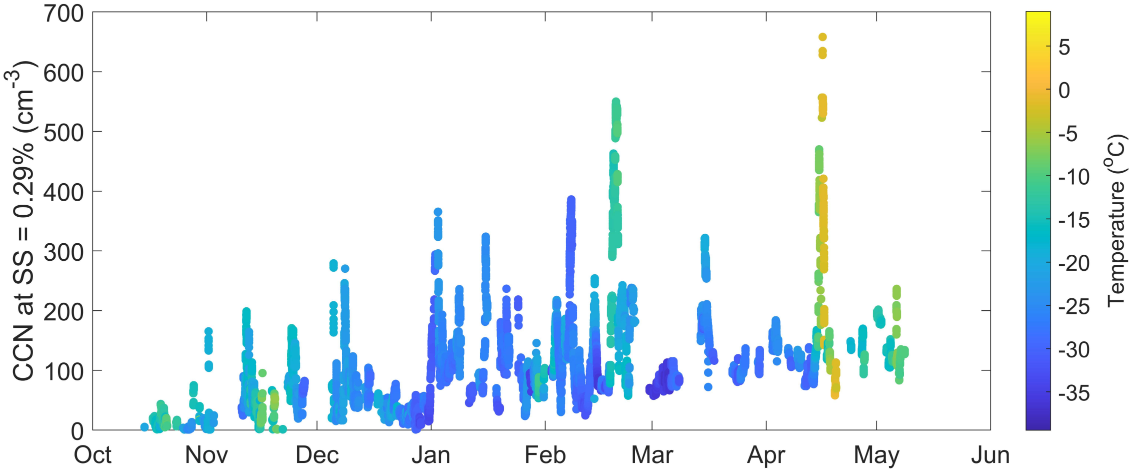

Supplementary Figure 13. Cloud condensation nuclei concentration during MOSAiC. Cloud condensation nuclei (CCN) concentrations at supersaturation (SS) = 0.29% during the MOSAiC year (Oct 15th 2019 – May 9th 2020) in 1- minute time resolution colored with temperature.

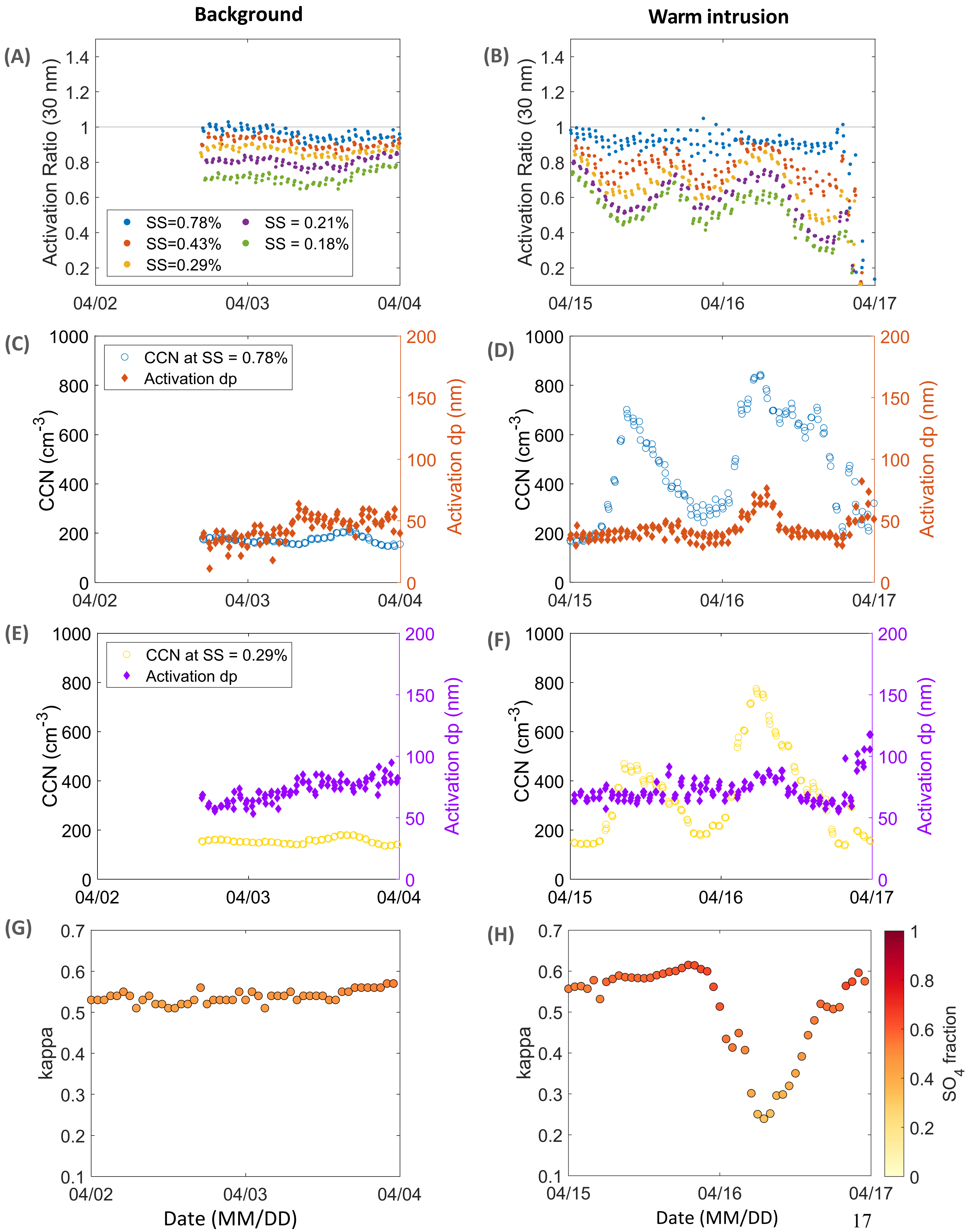
Background
Warm intrusion
(A)
(B)
Activation Ratio (30 nm)
SS=0.78%
SS=0.43%
SS=0.29%
SS = 0.21%
SS = 0.18%
04/02
04/03
04/04
04/15
04/16
04/17
(C)
(D)
CCN at SS = 0.78%
Activation dp
CCN (cm-3)
Activation dp (nm)
(E)
(F)
CCN at SS = 0.29%
(G)
(H)
kappa
SO4 fraction
Date (MM/DD)

Supplementary Figure 14. Cloud condensation nuclei properties. Activation ratio of cloud condensation nuclei (CCN) relative to > 30 nm particle concentrations during the (A) background and (B) warm intrusion period. CCN concentration at supersaturation (SS) = 0.78% (left axis, hollow blue circles) and critical activation diameter (right axis, orange diamonds) for the (C) background and (D) warm intrusion period. CCN concentration at SS= 0.29% (left axis, hollow yellow circles) and critical activation diameter (right axis, purple diamonds) for the (E) background and (F) warm intrusion period. Kappa value derived from aerosol chemical composition (see methods, subsection determination of aerosol hygroscopicity) in 1-hour averages for the (G) background and (H) warm intrusion period. We note that the CCN counter was not measuring between 02/04 at 0:00 and 17:00 due to maintenance procedures.

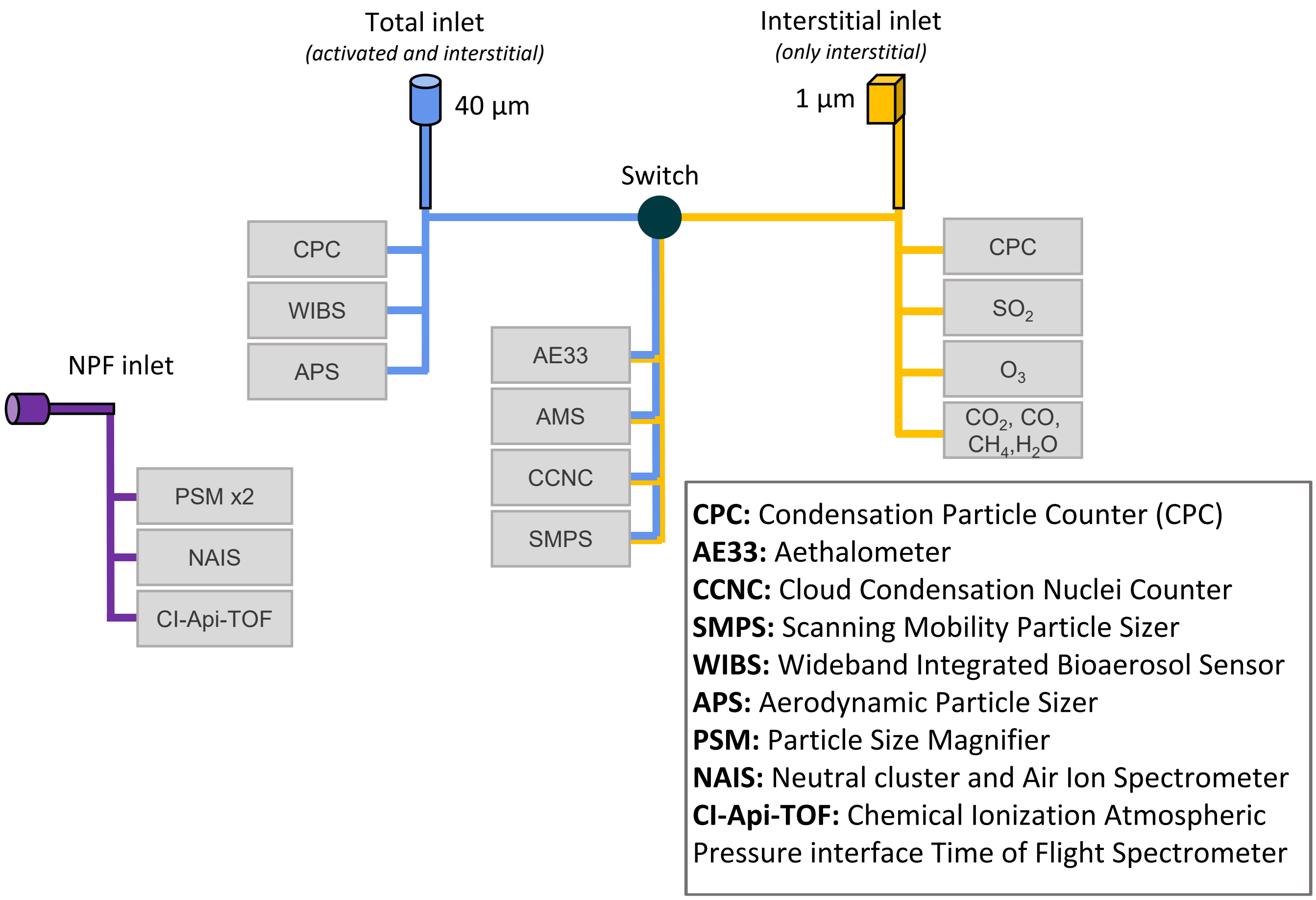

Supplementary Figure 15. Schematic of the instrumentation included in the Swiss container. Layout of the instruments inside the container. Aerosol particles and trace gases were sampled from three different inlets: (i) total inlet for sampling all particles and droplets up to 40 µm in diameter (blue inlet), (ii) an interstitial inlet equipped with a 1 µm cyclone for sampling particles that do not activate in cloud and fog (yellow inlet), and (iii) a new particle formation (NPF) inlet for sampling aerosol precursor gases, gas phase molecular clusters and small particles with a size of 1 – 40 nm (purple inlet). A switch rotated automatically every hour between the total and interstitial inlets for an alternate sampling from both inlets.

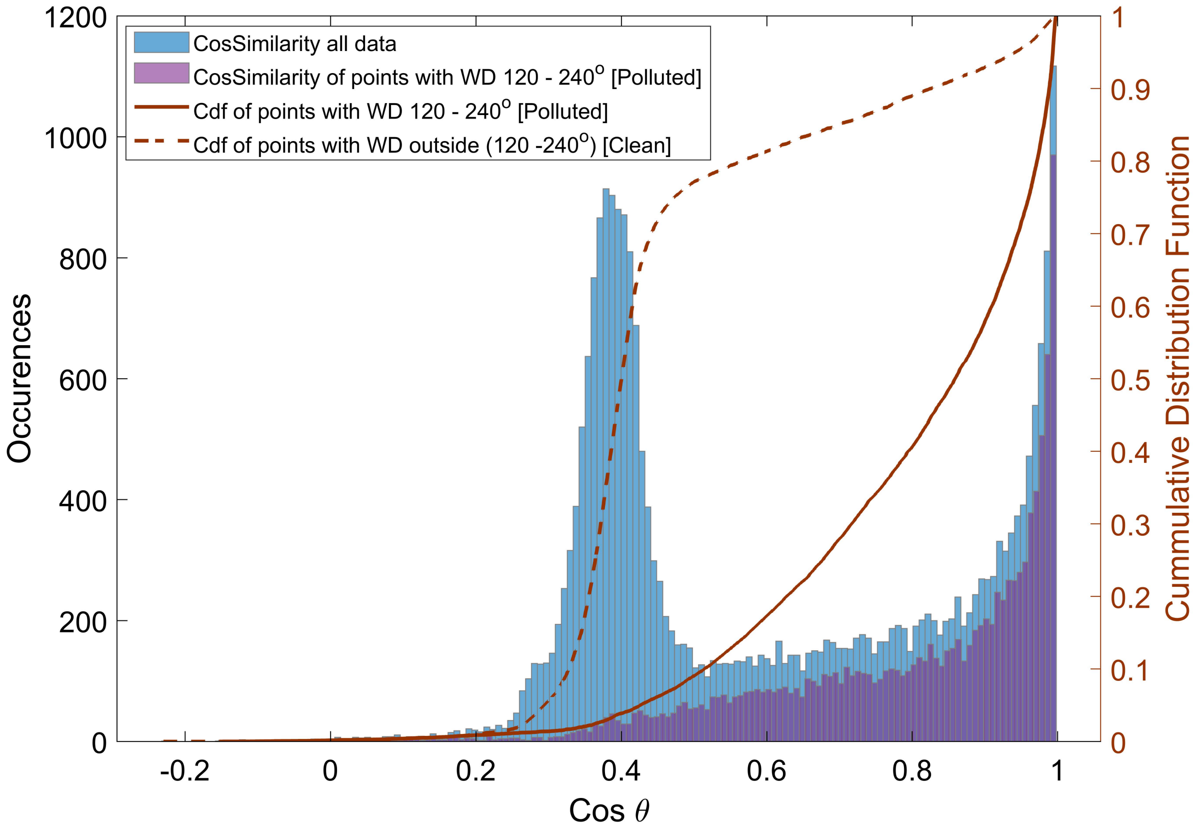

Supplementary Figure 16. Determination of aerosol mass spectrometer pollution flag threshold. (left axis) Histogram showing the cosine similarity between all point spectra and average ship exhaust spectrum (all data - in blue) and cosine similarity between all point spectra within the ship exhaust wind direction and average ship exhaust spectrum (polluted -in purple). (right axis) Cumulative distribution function of the cosine similarity between the point spectra affected by wind direction from ship exhaust and the average ship exhaust spectrum (polluted - solid line) and between the point spectra not affected by wind direction from ship exhaust and the average ship exhaust spectrum (clean - dashed line). In our case, a cos θ of 0.56 is considered the threshold above which data are considered contaminated by the ship exhaust.

Supplementary References

1. McDuffie, E. E. et al. A global anthropogenic emission inventory of atmospheric pollutants from sector- and fuel-specific sources (1970–2017): an application of the Community Emissions Data System (CEDS). Earth Syst. Sci. Data 12, 3413-3442, doi:10.5194/essd-12-3413-2020 (2020).
2. Jimenez, J. L. et al. Evolution of organic aerosols in the atmosphere. Science 326, 1525-1529, doi:10.1126/science.1180353 (2009).
3. Schmithüsen, H. Continuous meteorological surface measurement during POLARSTERN cruise PS122/3, Alfred Wegener Institute, Helmholtz Centre for Polar and Marine Research, Bremerhaven, doi: 10.1594/PANGAEA.935223 (2021).
